# A Smart Nanovector for Cancer Targeted Drug Delivery Based on Graphene Quantum Dots

**DOI:** 10.3390/nano9020282

**Published:** 2019-02-18

**Authors:** Daniela Iannazzo, Alessandro Pistone, Consuelo Celesti, Claudia Triolo, Salvatore Patané, Salvatore V. Giofré, Roberto Romeo, Ida Ziccarelli, Raffaella Mancuso, Bartolo Gabriele, Giuseppa Visalli, Alessio Facciolà, Angela Di Pietro

**Affiliations:** 1 Department of Engineering, University of Messina, Contrada Di Dio, I-98166 Messina, Italy; ccelesti@unime.it; 2 Department of Mathematical and Computer Sciences, Physical Sciences and Earth Sciences, Viale F. Stagno d’Alcontres, 98166 Messina, Italy; trioloc@unime.it (C.T.); patanes@unime.it (S.P.); 3 Department of Chemical, Biological, Pharmaceutical and Environmental Sciences, University of Messina, Viale Annunziata, I-98168 Messina, Italy; sgiofre@unime.it (S.V.G.); robromeo@unime.it (R.R.); 4 Laboratory of Industrial and Synthetic Organic Chemistry (LISOC), Department of Chemistry and Chemical Technologies, University of Calabria, Via Pietro Bucci 12/C, 87036 Arcavacata di Rende (CS), Italy; idaziccarelli@gmail.com (I.Z.); raffaella.mancuso@unical.it (R.M.); bartolo.gabriele@unical.it (B.G.); 5 Department of Biomedical and Dental Sciences and Morphological and Functional Images, University Hospital of Messina, Via Consolare Valeria, 1, 98100 Messina, Italy; gvisalli@unime.it (G.V.); afacciola@unime.it (A.F.); adipietr@unime.it (A.D.P.)

**Keywords:** graphene quantum dots, drug delivery systems, anticancer therapy

## Abstract

Graphene quantum dots (GQD), the new generation members of graphene-family, have shown promising applications in anticancer therapy. In this study, we report the synthesis of a fluorescent and biocompatible nanovector, based on GQD, for the targeted delivery of an anticancer drug with benzofuran structure (BFG) and bearing the targeting ligand riboflavin (RF, vitamin B2). The highly water-dispersible nanoparticles, synthesized from multi-walled carbon nanotubes (MWCNT) by prolonged acidic treatment, were linked covalently to the drug by means of a cleavable PEG linker while the targeting ligand RF was conjugated to the GQD by π–π interaction using a pyrene linker. The cytotoxic effect of the synthesized drug delivery system (DDS) GQD-PEG-BFG@Pyr-RF was tested on three cancer cell lines and this effect was compared with that exerted by the same nanovector lacking the RF ligand (GQD-PEG-BFG) or the anticancer drug (GQD@Pyr-RF). The results of biological tests underlined the low cytotoxicity of the GQD sample and the cytotoxic activity of the DDS against the investigated cancer cell lines with a higher or similar potency to that exerted by the BFG alone, thus opening new possibilities for the use of this drug or other anticancer agents endowed of cytotoxicity and serious side effects.

## 1. Introduction

Nano-Oncology, the application of nanotechnology for the treatment of cancer, has achieved outstanding growth over the last decade [1]. Engineering nanomaterials for cancer therapy and diagnosis have shown the potential to revolutionize the cancer treatment by developing new theranostic nanoparticles for simultaneous imaging and therapy [2,3]. The current cancer research is focused on developing drug-targeted nanoparticles with the ability to overcome the mechanisms of drug resistance and to achieve a more efficient local drug delivery for poorly vascularized regions of tumors with minimal damage to normal tissues [4,5,6]. Due to their small size and large surface area, these nano-sized drugs show enhanced bioavailability and additional ability to cross human cell membranes, thus improving the biodistribution of anticancer agents. The cell uptake of the nanoparticles mainly occurs through endocytosis which leads to their engulfment in membrane invaginations and the subsequent formation of endocytic vesicles which transport these particles in intracellular compartments. The cellular fate of nanoparticles designed for targeting cancer cells is strongly influenced by the tumor microenvironment and, in particular, by the poor lymphatic drainage which leads to elevated interstitial fluid pressures and by pH which affects the nanoparticle-cell interactions and the nanoparticle uptake [7]. Complex macromolecules, such as proteins and nucleic acids, as well as poorly soluble synthetic drugs, have been dispersed in nanoparticle-based formulations maintaining their stability and chemotherapy efficacy, improving the drug solubility, reducing their toxicity, and also providing site-specific targeting [8,9]. However, although promising results have been reported with nanoparticles based on anticancer therapy, their inherent toxicity and unfavorable biological distribution are still major restrictions that hinder the clinical translation of advanced nanoplatforms [10,11]. Thus, new formulations are needed to increase the nano-drug efficiency while reducing systemic side effects and minimizing damage to surrounding normal tissues.

Graphene-based nanomaterials, such as fullerenes, graphene oxide, and carbon nanotubes, have attracted great interest in the biomedical field, owing to their extraordinary chemical, physical, and mechanical properties [12,13,14,15]. Even if several in vitro and in vivo studies have demonstrated the physiological ability of functional graphene nanomaterials to promote interfacial biointeractions with mammalian cells as well as their potential toxicity in different tissues and organs, the toxicity risks of graphene-based materials are strongly dependent on their surface functionalization and size [16]. The next generation members of graphene-family, the graphene quantum dots (GQD), have shown even more promising applications in cancer treatments, pulse sensing, and prosthetics [17,18,19]. When compared with other carbon-based nanomaterials and to conventional quantum dots (QD), GQD originating from graphene or various organic sources have some particularly unusual chemical/physical properties, such as low toxicity, chemical inertness, water solubility, and biocompatibility, which make them ideal nanocarriers for drug delivery [20]. Moreover, the quantum confinement, which confers fluorescence to these nanostructures, allows the simultaneous detection and treatment of cancer cells, making them suitable platforms for theranostic purposes [21]. GQD have proved to improve the chemotherapy efficacy of anticancer agents acting at the nuclear level, such as doxorubicin or cisplatin, by accelerating their nuclear accumulation and enhancing their DNA cleavage activity [22]. The presence of different reactive groups on the graphene surface allows the multimodal covalent and non-covalent conjugation with drugs, targeting ligands, and polymers, in order to improve their pharmacological profile both in vitro than in vivo [23]. The targeting strategy, based on the carrier surface functionalization with ligands, that can be selectively recognized by receptors present on the surface of cancer cells, can effectively minimize the systemic toxicity typically associated with conventional cancer therapy [24].

**Figure 1:**
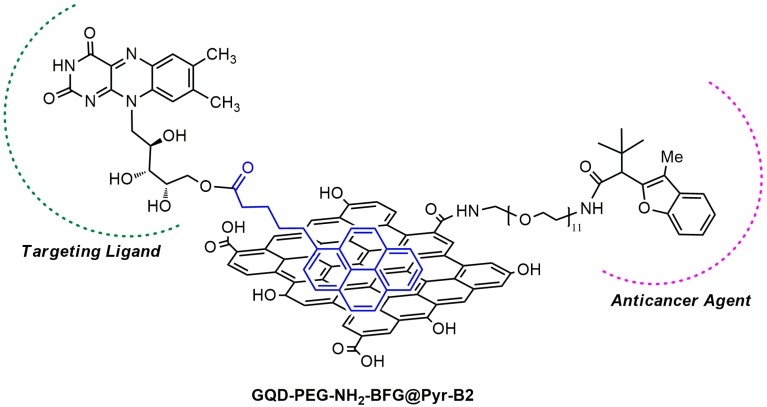
Cancer targeted drug delivery system (DDS) based on graphene quantum dots (GQD).

The purpose of this work was the development of a cell traceable and biocompatible cancer-targeted drug delivery system (DDS), based on GQD, and bearing an anticancer agent with benzofuran structure and the targeting ligand riboflavin (vitamin B2) (Figure 1).

The GQD used in this study were produced by acid treatment and chemical exfoliation of multi-walled carbon nanotubes (MWCNT), where the presence of multiple defects in the graphene layers allows the production of graphene fragments containing many reactive oxygen functional groups and endow of water dispersibility [25].

The anticancer drug chosen for this study, the methyl 3,3-dimethyl-2-(3-methyl-2,3-dihydrobenzofuran-2-yl)butanoate (BFG), belongs to a series of benzofuran-2-acetic ester derivatives, recently synthesized by us [26,27], which is able to inhibit growth and induce apoptosis in breast cancer cells by enhancing p21^Cip/WAF1^ gene expression in p-53 in an independent manner [28]. The drug was covalently conjugated to the surface of GQD through a cleavable PEG linker which can be activated specifically inside human cells. The surface modification with PEG is generally considered the preferred method to improve the biocompatibility and to mask nanoparticles from immune recognition [29]. 

The targeting ligand chosen in this study, riboflavin (RF), is an important coenzyme in energy metabolism. The presence of RF receptors overexpressed in human cancer cells from prostate and breast cancers have shown to improve the cancer cell uptake of RF and RF conjugates by receptor-mediated endocytosis [30]. Riboflavin-presenting dendrimers have shown to efficiently deliver methotrexate to Hela cells via receptor-mediated internalization [31].

In this work, RF was conjugated to the surface of GQD by taking advantage of the stable π–π interaction of this nanomaterial with pyrene derivatives. In addition to its targeting ability, this vitamin is particularly interesting for the design of an ideal DDS due to its water solubility, biocompatibility, and for intrinsic fluorescence which allows its in vitro traceability [32].

The results of in vitro biological tests performed on the GQD-based systems have shown the great biocompatibility of the synthesized nanocarrier and the good ability of the developed DDS to increase drug-cells interactions, causing cytotoxicity in all the investigated cell lines. The high water dispersibility of the riboflavin conjugated DDS has shown the potential to overcome the critical limitations of the currently used anticancer drugs.

## 2. Materials and Methods 

### 2.1. Materials

All reagents and starting materials were purchased from commercial suppliers and were used without further purification. The MWCNT used for GQD synthesis were synthesized by catalytic chemical vapor deposition (CCVD) starting from isobutane as a carbon source and using Fe/Al_2_O_3_ as a catalyst. The purification procedure gave pristine MWCNT with >95% purity [33]. GQD were obtained from MWCNT by treatment with a 1:3 ratio mixture of HNO_3_/H_2_SO_4_, following a previously reported procedure [23]. Benzofuran G (BGF) was synthesized as previously reported [26,27,28].

### 2.2. Chemical, Physical, and Morphological Characterization

^1^H NMR spectra were recorded on a Varian instrument operating at 500 MHz, and the chemical shifts were reported in ppm relative to TMS as standard. The thin-layer chromatography was done using Merck silica gel 60-F254 pre-coated aluminum plates, and the preparative separations were performed by flash chromatography with Merck silica gel of 0.063–0.200 mm and 0.035–0.070 mm. The morphology of GQD was investigated by high-resolution transmission electron microscopy (HRTEM) using an analytical electron microscope JEOL JEM 2010 (LaB_6_ electron gun), operating at 200 kV, equipped with a Gatan 794 Multi-Scan CCD camera for the digital imaging. The samples investigated for HRTEM were obtained by placing a few drops of GQD, dispersed in isopropanol, on the 400 mesh holey carbon-coated copper grids. Thermogravimetric investigations were performed on a TA Q500 instrument, at 10 °C/min, from 100 to 1000 °C, inargon. Zeta potential measurements and titration analyses were accomplished using the Zeta sizer 3000 instrument (Malvern). The titration analyses were carried out in the pH value range of 6–8. The Infrared spectra were obtained using the Fourier-Transform Infrared (FT-IR) spectrometer Perkin-Elmer 2000 with the KBr pellets method. The measurements of photoluminescence (PL) were performed using a NanoLog modular spectrofluorometer Horiba under excitation by a xenon lamp at room temperature. GQD-based samples were prepared at a concentration of 100 ng/mL. Atomic force microscopy (AFM) images of samples surface were acquired using an NT-MDT NTEGRA Spectra microscope and employing a Si-cantilever working in semi-contact mode. The Micro Raman measurements were obtained in a reflection mode by exciting the samples with a solid-state laser at λ_exc_ = 470 nm. The high resolution mass spectrometry (HRMS) spectrum of Pyr-RF sample was performed with a Finnigan MAT 95 instrument, EI: 70 eV, R:10,000. The purity of Pyr-RF sample was determined by HPLC (Varian ProStar 325) equipped with a C-18 bounded-phase column (Waters, XTerra C18 MS, 3.5 µm, 4.5 × 50 mm). Gradient elution was performed with acetonitrile and water as a mobile phase and was monitored at 254 nm.

### 2.3. Synthesis of GQD

GQD were synthesized from pristine MWCNT by treatment with a solution of HNO_3_/H_2_SO_4_ in a 1:3 ratio. The suspension was placed in a reaction vessel with a water condenser in an ultrasonic bath at 60 °C for 4 days, under reflux. After dilution with deionized water, the mixture was filtered using a 0.1 µm Millipore membrane under vacuum. Then, the filtrate was treated with a NaOH solution until neutral pH and subjected to centrifugation at 3000 rpm. The obtained mixture was diluted with deionized water and purified using a dialysis bag with a molecular weight (MW) of 12,000 Da. Little amounts of the resulting material were dried at 60 °C, under vacuum, and used for characterizations. Titration analysis was used to quantify the number of acidic groups present on the surface of GQD, which was found to be 2.37 mmol/g.

### 2.4. Synthesis of GQD-PEG

A solution of GQD (6 mg) in DMF (10 mL) was treated with *N*-(3-dimethylaminopropyl)-*N*′-ethylcarbodiimide hydrochloride (EDC·HCl, 3 mg, 0.016 mmol) and triethylamine (ETA, 2.2 µL, 0.016 mmol), and the mixture was left to stir at room temperature for 15 min. Then, hydroxy benzotriazole (HOBt, 2.2 mg, 0.016 mmol) and a catalytic amount of 4-dimethylaminopyridine (DMAP) were added to the mixture which was left to stir for 1 h. The suspension was then treated with 10.3 mg of *O*-(2-aminoethyl)-*O*′-[2-(Boc-amino)ethyl]decaethylene glycol (0.016 mmol) and was left to stir at room temperature for 4 days. The obtained residue was diluted with deionized water and purified using dialysis bags with an MW of 12,000 Da for 8 h. The Boc-protecting group was subsequently removed by treating the mixture with HCl (4 M) in dioxane for 1 h at room temperature, affording the sample GQD-PEG. The NH_2_ loading was calculated by Kaiser test and was found to be 0.9 mmol g^−1^. The degree of functionalization was measured by TGA under argon atmosphere, after drying a known amount of sample, under vacuum, at 60 °C.

### 2.5. Synthesis of GQD-PEG-BFG

A solution of potassium tert-butoxide (0.046 mmol) in THF (5 mL, containing 0.2% of H_2_O) was left to stir in the air for 1 min at room temperature. Then, BFG (6 mg, 0.023 mmol) and dispersion of GQD-PEG (6 mg) in pure water were added, and the mixture was left to stir at room temperature for 1 h. After removal of THF, under vacuum, the mixture was diluted with water and purified using a dialysis bag (MW of 12,000 Da) for 8 h. A known amount of the resulting suspension was dried, under vacuum, at 60 °C for the chemical and physical characterizations. The amount of drug present on the GQD was evaluated by TGA analysis and was found to be 21 wt%.

### 2.6. Synthesis of Pyr-RF

A solution of riboflavin (15 mg, 0.04 mmol) in CH_2_Cl_2_ (20 mL) was treated with EDC·HCl (7.6 mg, 0.04 mmol) and ETA (5 µL, 0.4 mmol), and the solution was left to stir at room temperature for 15 min. DMAP (4.8 mg, 0.04 mmol) and HOBt (4 mg, 0.04 mmol) were then added, and the mixture was stirred for an additional 1 h. Then, 12 mg of 1-pyrene butyric acid (0.04 mmol) was added, and the suspension was left to stir for 5 days at room temperature. The mixture was then extracted with CH_2_Cl_2_ (3 × 50 mL), and the organic layers were dried using MgSO_4_ and concentrated, under vacuum, affording the crude product. The purification by medium pressure liquid chromatography (MPLC) on a silica gel column using a mixture of CH_2_Cl_2_/MeOH (98:2) as eluent afforded the pure conjugated compound 5-(7,8-dimethyl-2,4-dioxo-3,4-dihydrobenzo[g]pteridin-10(2H)-yl)-2,3,4-trihydroxypentyl-5-(pyren-1-yl)pentanoate (Pyr-RF) with purity of ≥98% as determined by HPLC analysis (see ESI for NMR and Mass Spectrometry data and spectra).

### 2.7. Synthesis of GQD-PEG-BGf@Pyr-RF and GQD-Pyr-RF

A solution of GQD-PEG-BFG (10 mg) or GQD (10 mg) in phosphate buffer solution (PBS) at pH 7.4 (5 mL) was treated with a solution of Pyr-RF (10 mg) dissolved in 5 mL of PBS at room temperature for 5 days. The nanosystems were then purified using dialysis bags of MW 12,000 Da for 8 huntil no amount of unbound Pyr-RF was detected in the washing solutions by NMR. The amount of Pyr-RF bound to the GQD surface was found to be 19.2 wt% for GQD-PEG-BGf@Pyr-RF and 8.5% for GQD-Pyr-RF. Known amounts of the resulting materials were dried at 60 °C, under vacuum, for the chemical and physical characterizations.

### 2.8. Biological Assays

The cytotoxicity of the GQD, GQD-PEG, GQD-PEG-BFG, GQD@Pyr-RF, GQD-PEG-BFG@Pyr-RF, and BFG were detected by measuring the reduction of the dye 3-(4,5-dimethylthiazol-2yl)-2,5-diphenyltetrazolium bromide (MTT) in three different cancer cell lines: laryngeal cancer cell line (HEp-2), human lung epithelial cancer cell line (A549), and human colorectal adenocarcinoma cell line (HT-29). Briefly, by using 96-well plates, the confluent monolayers of the different cell lines were treated with suspensions of the synthesized GQD-based nanosystems in cell medium (containing 2% of FCS) for 24 h. The assayed concentrations were 10, 25, 50, 100, and 200 μg mL^−1^ for GQD-PEG-BFG@Pyr-RF, corresponding to 2.1, 5.25, 10.5, 21, and 42 μg mL^−1^ does of BGF assayed in the positive controls. For the GQD-PEG-BFG, the assayed doses were calculated to assess the same drug concentrations while for GQD, GQD-PEG, and GQD@Pyr-RF, the examined doses were 10, 25, 50, 100, and 200 μg mL^−1^. The cells kept in the same medium with PBS were used as negative control. After treatment, the cells were washed with PBS for three times, and a solution of MTT (0.5 mg mL^−1^) was added to the cell medium without phenol red and incubated for 3 h at 37 °C. Then, a mixture of 50 mM HEPES at pH 8.0 and ethanol (1:9, v/v) was added in order to solubilize the resulting violet colored formazan crystals. The absorbance was recorded at 540 nm using a microtiter plate reader (Tecan Italia, Milan-Italy). The obtained values were compared with those obtained by the negative control whose percentage of live cells was considered to be 100. The biological tests were all performed in triplicate, and the replicates were calculated as means ± SD.

## 3. Results and Discussion

### 3.1. Synthesis and Characterization of GQD

**Figure 2:**
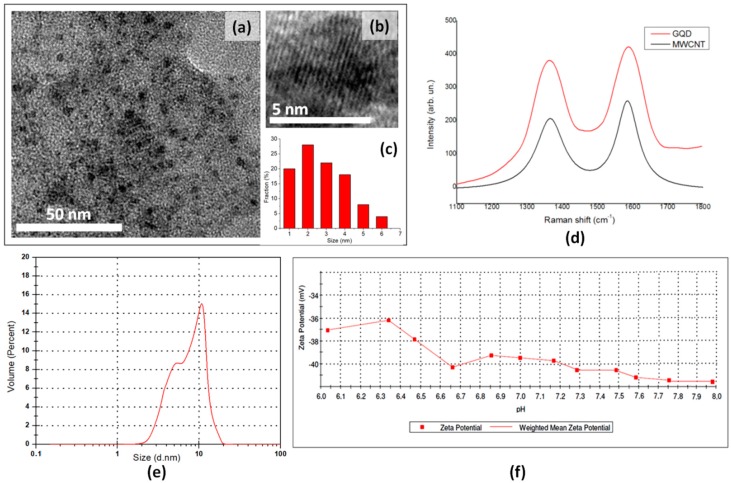
(**a**) Representative TEM image of the as-prepared GQD; (**b**) high-resolution TEM image showing the crystal structure of an individual GQD;(**c**) size distribution of GQD derived from TEM images; (**d**) Raman spectra of GQD and MWCNT; (**e**) volume-weighted size distribution of GQD dispersions in deionized water; (**f**) isoelectric titration graph of the GQD sample in the pH range of 6–8 (evaluated as dependence of zeta potential on the pH of the medium for aqueous solutions of GQD).

The carbon source and the oxidation method used to synthesize GQD are pivotal factors able to affect the physicochemical and morphological features of the synthesized nanomaterials as well as their biocompatibility. Small graphene fragments, containing many reactive oxygen functional groups and good dispersion stability in water, were obtained by acidic oxidation and chemical exfoliation of MWCNT, following a procedure previously reported by us [23]. The presence of multiple defects in the MWCNT graphene layers allows the introduction of many oxygen-containing functionalities onto the surface and edge of graphene fragments and increases the active sites for their organic modification. The synthesized GQD were investigated by XRD, DLS, UV, PL, Raman, and HRTEM analyses (Figure 2).

The representative TEM images of the as-prepared GQD show monodisperse particles with a size distribution between 1 and 6 nm (Figure 2a) and a weighted size distribution centered at 2.78 nm, as evaluated using statistical calculations performed on more than 200 dots (Figure 2c). The HRTEM image shows the crystalline graphene structure of an individual GQD (Figure 2b). The presence of thisstructure, which opens the possibility for organic surface modification with biologically active agents, together with their uniform and small size, make these nanomaterials ideal nanoplatforms for drug delivery. The XRD spectrum of GQD sample, compared with that of precursor MWCNT, does not show any diffraction signals in the whole range 2-theta, while MWCNT shows the presence of the (002) peak. This peak, which is generally considered a measure of the interplanar spacing for two neighboring graphene layers, is totally absent in the GQD sample and suggestsa single layer structure after acidic exfoliation (Figure S1, ESI) [34].

In Figure 2d, the Raman spectra of GQD and of MWCNT are shown. Both spectra show the D-band (*ca.* 1360 cm^−1^) and the G-band (*ca.* 1590 cm^−1^), which represent the Raman fingerprint of carbon nanostructures. While D-band is attributed to the disorder in the sp^2^ hybridized carbon, the G-band is associated with the first-order scattering of the stretching vibration mode E_2g_ observed for the sp^2^ carbon [35]. The relative intensity ratio of D and G bands (I_D_/I_G_ ratio) showed a value of 1.033 for the pristine MWCNT and a value of 1.145 for the GQD sample, thus further proving the loss of long-range order after the acidic treatment.

The volume-weighted DLS measurements confirm the small size of the synthesized nanomaterial, showing a maximum of volume weighted percent at 10.9 nm, with a shoulder at 5.3 nm (Figure 2e). The value of polydispersity index (PDI) of 0.1, as evaluated by DLS analysis, further confirm the narrow particle size distribution.

The strong oxidative treatment together with the long sonication time have proved to afford GQD with many oxygen-containing groups charged onto the graphene surface. Thetitration analysis performed on GQD, by evaluating the dependence of their zeta potential values on the pH of the medium, showed a greater amount of acidic group (2.37 mmol/g) with respect to that observed for the oxidized MWCNT sample (1.8 mmol/g), reported previously, and obtained after only 6 h of oxidative treatment [14]. This oxidative treatment also enhances the GQD dispersion stability in water, thus affording the biocompatibility of nanomaterials and making it easier to be transported along the physiological milieu and then its excretion [36]. The GQD dispersion stability in water was evaluated by calculating the zeta potential values of the synthesized nanomaterials in the pH range of 6.0–8.0 (Figure 2f). Nanoparticles have shown to acquire a more negative surface charge when increasing the pH of the medium, showing zeta potential values always lower than −30 mV. As shown in the literature, nanoparticles, endowed with zeta potentials values greater than +30 mV or more negative than −30 mV, repel each other and show no tendency to aggregate, thus affording high stability in water [37].

The optical properties of GQD were evaluated by UV and photoluminescence (PL) analyses (Figures S2 and S3, ESI). The UV spectrum of the GQD sample (Figure S3) shows the representative absorption band at 250 nm related to the π–π* (bonding-antibonding) transition of the sp^2^ aromatic domains. The PL measurements confirm the homogeneous distribution of the GQD particle size (Figure S3, ESI), a seven on exciting the GQD water dispersions at the wavelengths range of 330–370 nm, a strong peak at 560 nm is always observed.

### 3.2. Synthesis of GQD-PEG-BFG

**Scheme 1:**
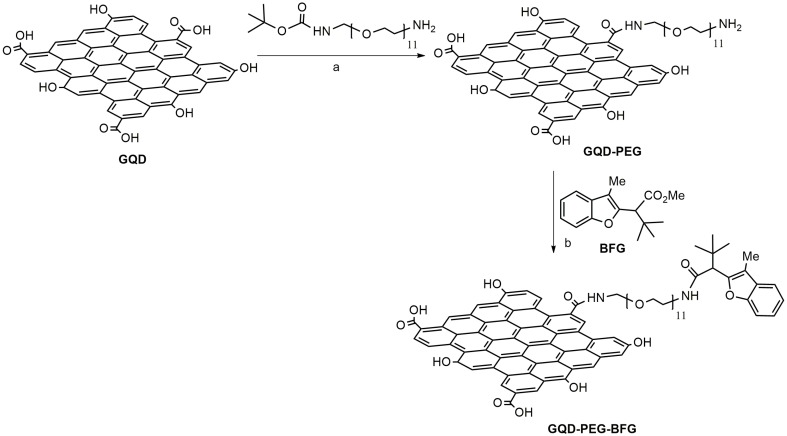
Synthesis of GQD-PEG-BFG. *Reagents and conditions*: (**a**) Boc-NH-PEG-NH_2_, EDC⋅HCl, HOBt, ETA, DMAP, DMF, 4 d, r.t., then, HCl 4 M, dioxane, 1 h, r.t.; (**b**) BFG, KO*t*Bu, air. H_2_O/THF, 1 h, r.t.

The anticancer drug, BFG, was loaded to the surface of GQD by means of the bidentate linker 2,2-(ethylenedioxy)bis(ethylamine) which is protected at a single amine functionality with the tert-butyloxycarbonyl (BOC) group. The coupling reaction between the carboxylic functionalities present on the GQD surface and the free amino group of the PEG linker was accomplished using one equivalent of DMAP and EDC·HCl/HOBt as coupling agents. The successive BOC-deprotection of the second amino group gave the amino-functionalized nanosystem GQD-PEG, whose amine loading was found to be 0.9 mmol g^−1^, as calculated by Kaiser test. The nanosystem was then conjugated with the anticancer drug, BFG, by tert-butoxide-assisted amidation reaction between the amino group of the nanosystem and the ester functionality of the benzofuran derivative following a procedure reported by Kim et al. [38] (Scheme 1).

**Figure 3:**
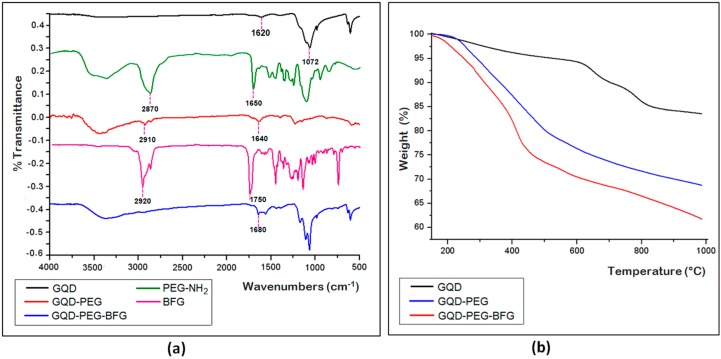
(**a**) FTIR spectra of GQD, GQD-PEG, GQD-PEG-BFG, PEG-NH_2_, and BFG; (**b**) TGA curves for GQD, GQD-PEG, and GQD-PEG-BFG. The experiments were performed under an argon atmosphere.

The success of the reaction coupling between GQD and PEG linker and of the subsequent drug loading to the surface of GQD were investigated by TGA and by FTIR spectroscopy (Figure 3a,b).The FTIR spectrum of GQD shows two peaks at 1620 and 3450 cm^−1^, which are related to the vibrations of C=O and O−H bonds, respectively. The additional peak at 1072 cm^−1^ is ascribable to the C−O alkoxy group (Figure 3a). These data further confirm the presence of many oxygen-containing groups on the GQD surface. The FTIR spectrum of the pegylated sample (GQD-PEG) shows the representative additional peak at 1640 cm^−1^, which is related to the C=O stretching of the newly formed amide bond and a broader absorbance at 3400–3460 cm^−1^ ascribable to the O–H stretching, due to the ethylene glycol groups. In addition, a wide absorbance at 3050–3200 cm^−1^ related to the N–H bending, and the presence of a peak at 2910 cm^−1^ due to C–H stretch of PEG chain, can be observed. The peaks at 1650 and 2870 cm^−1^ present in the FTIR spectrum of PEG-NH_2_ sample and due to the N-H bending and C-H stretching, respectively, are considerably weaker in the conjugated sample GQD-PEG, thus further proving the formation of the amide bond between the free amino group of PEG-NH_2_ and the carboxyl groups of GQD. The FTIR spectrum of the drug-conjugated sample (GQD-PEG-BFG) shows the presence of an additional peak at 1680 cm^−1^, ascribable to the C=O stretching of the amide bond formed after the drug conjugation of the functionalized GQD with BFG. The FTIR spectrum of the anticancer agent, BFG, shows the presence of a peak at 2920 cm^−1^ due to the C–H stretching of the aliphatic chain of BFG and a peak at 1750 cm^−1^ due to the C=O stretching of the ester functionality. The disappearance of this latter peak demonstrates the effectiveness of the conjugation reaction between the drug and the free amino groups present in the nanosystem.

The TGA curves of the functionalized GQD and of precursors GQD, performed under an inert atmosphere, show an increase of weight losses for both functionalized samples, whose amounts can be related to the increased amount of organic moiety present on the surface of GQD surface (Figure 3b). The different profiles of TGA curves of conjugated compounds further confirm the deep chemical modifications occurred on the nanostructures after the functionalization processes and are in agreement with the TGA curves of PEG-NH_2_ and BFG, showing a quite different profile with a narrow degradation range at 250–400 °C and 180–280 °C for PEG-NH_2_ and BFG, respectively (Figure S4, ESI). The drug loading on the GQD surface was measured as the difference between the amount of BFG added in the coupling reaction mixture and the amount of drug recovered in the solutions filtered from the dialysis bag and also by TGA analysis at 500 °C under argon atmosphere. In both cases, this value was found to be 21.6 wt%.

### 3.3. Synthesis of GQD-PEG-BFG@Pyr-RF and GQD@Pyr-RF

**Scheme 2:**
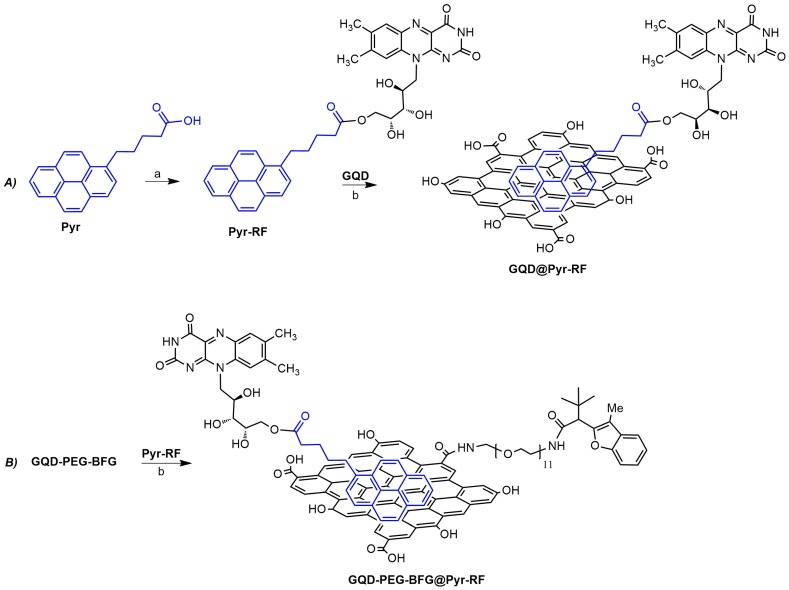
Synthesis of GQD@Pyr-RF and GQD-PEG-BFG@Pyr-RF. *Reagents and conditions*: (**a**) Riboflavin, EDC·HCl, ETA, DMAP, HOBt, CH_2_Cl_2_,1 h, r.t., then 1-pyrene butyric acid, 5 d, r.t.; (**b**) GQD-PEG-BFG or GQD, Pyr-RF, PBS (pH 7.4), 5 d, r.t.

Graphene-based materials can form stable π–π interactions with molecules with short to highly extended π system [39]. We exploited this methodology to load the targeting ligand riboflavin (RF) to the graphene surface by means of aromatic interaction between the pyrene derivative and the sp^2^ domains of GQD. RF was linked to the pyrene moiety by coupling reaction between the free carboxylic groups of 1-pyrene butyric acid (Pyr) and the primary alcoholic group of the vitamin using the coupling agents EDC/HOBt and affording the Pyr-RF conjugate in quantitative yield (Scheme 2). The compound was purified using MPLC chromatography and was characterized through NMR spectroscopy. The ^1^H NMR analysis of the Pyr-RF conjugate revealed the presence of the aromatic protons of pyrene moiety in the range of 7.68–7.83 ppm, the presence of the aromatic protons of riboflavin at 6.40 and 6.85 ppm, and the representative peak of the methylene protons of the newly formed ester bond at 3.89–4.08 ppm. 

GQD were conjugated with the targeting module Pyr-RF by taking advantage of the stable π–π interaction of these nanomaterials with pyrene derivatives. In order to deeper investigate the targeting ability of RF, this vitamin was charged in the drug-conjugated sample (GQD-PEG-BFG) than in the GQD sample (Scheme 2). The RF loadings were carried out in basic buffer solution (PBS) at pH 7.4, in order to obtain stable π–π stacking interactions with the surface of GQD [40].

The amounts of RF loaded on the GQD@Pyr-RF and GQD-PEG-BFG@Pyr-RF nanosystems were assessed by the difference between the amount of ligand mixed with the GQD-based materials and the amount of compound present in the washing solutions after dialysis treatment. The loading values were found to be 9 and 20 wt% for GQD@Pyr-RF and GQD-PEG-BFG@Pyr-RF, respectively. The obtained values are in complete agreement with the data calculated by TGA analysis (Figure S7, ESI). The percentages of weight losses calculated under an inert atmosphere at 500 °C were found to be 8.5 and 19.2 wt% for GQD@Pyr-RF and GQD-PEG-BFG@Pyr-RF, respectively.

**Figure 4:**
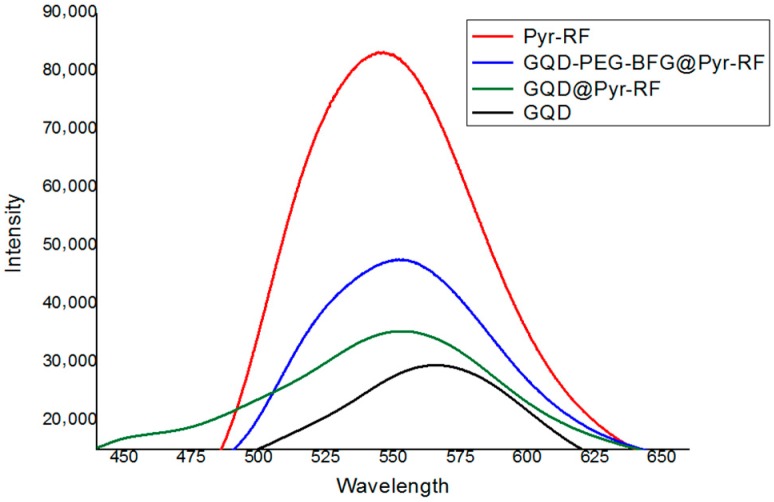
PL spectra of GQD (black line), GQD@Pyr-RF (green line), GQD-PEG-BFG@Pyr-RF (blue line), and Pyr-RF (red line) in water, at the excitation wavelength of 360 nm. The samples were tested at a concentration of 100 ng/mL.

The presence of a stable interaction between the targeting module Pyr-RF and the GQD surface was also proved by evaluating the fluorescence properties of GQD@Pyr-RF and GQD-PEG-BFG@Pyr-RF and comparing their PL spectra with those of GQD and of the unbound Pyr-RF samples at the excitation wavelength of 360 (Figure 4). As reported, some small molecules exhibit fluorescence quenching ability after their π–π stacking with the surface of graphene-based materials [41]. The PL spectra of the GQD-based samples conjugated with the Pyr-RF module reveal a quenching phenomenon of the vitamin fluorescence, which is ascribable to an electron transfer process triggered by π–π stacking interactions between the aromatic rings of RF and the p-cloud of GQD. This decrease in the fluorescence intensity together with the redshifts of conjugated samples from 566 (in the GQD sample) to 551 and 553 nm in the GQD@Pyr-RF and GQD-PEG-BFG@Pyr-RF), respectively, are indicative of the stable chemical interaction between the targeting module and the GQD-based nanomaterial.

**Figure 5:**
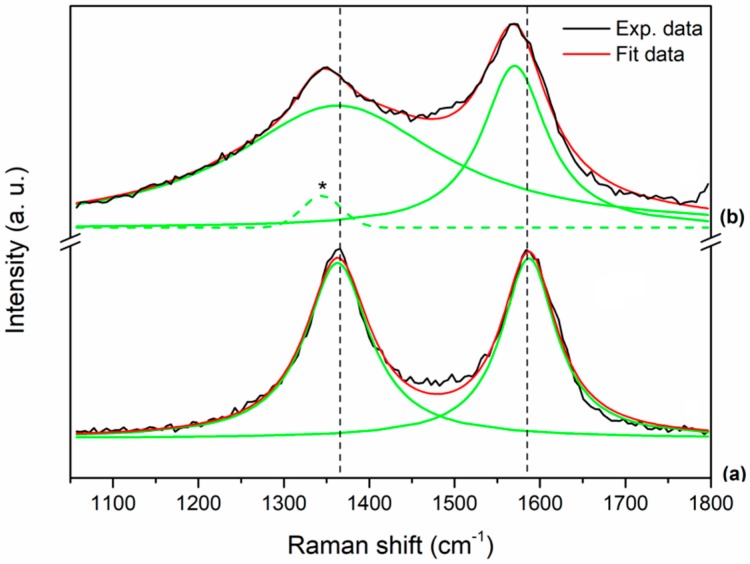
Raman spectra of GQD (curve a) and of the DDS GQD-PEG-BFG@Pyr-RF (curve b) samples. Lorentzian peaks (green lines) are used to reproduce the Raman spectrum.

The Raman spectra of the final DDS GQD-PEG-BFG@Pyr-RF, compared with the reference sample GQD, show the distinctive D-band (*ca.* 1360 cm^−1^) and G-band (*ca.* 1590 cm^−1^) for both the materials encountered for sp^2^ carbon nanostructures (Figure 5). After the functionalization process, the D-band and G-band of the final DDS appear broadened with slightly blue-shift. The D-band shifts from 1363 cm^−1^ in GQD (Figure 5, curve a) down to 1345 cm^−1^ in GQD-PEG-BFG@Pyr-RF (Figure 5, curve b), while the G-band shifts from 1587 cm^−1^ in GQD down to 1570 cm^−1^ in the functionalized sample. In order to understand the effects of the functionalization process in GQD, the Raman spectrum of the GQD-PEG-BFG@Pyr-RF was fitted using three peaks: the G-band is fitted with a Lorentzian peak, while the function used to fit the D-band consists of a Lorentzian peak and a Gaussian peak (dotted green line and marked in curve b of Figure 5). This new Raman mode, located at lower frequencies compared to the D-band, is related to the functionalization process of the sample GQD [42,43]. The D/G integrated intensity ratio (I_D_/I_G_), commonly used to monitor the sp^2^ defect density in the carbon lattice (I_D_/I_G_ ratio), increases from 1.14 in GQD to 2.01 in GQD-PEG-BFG@Pyr-RF, thus indicating that after the functionalization processes, the sp^2^ carbon defects density increases too. The observed ratio is in complete agreement with the results obtained for similar pyrene modified graphene-based materials and can be explained by the charge transfer of pyrene moiety to the graphene matrix after π–π interaction. This transfer can change the electronic structure of graphene by inducing probably n-type doping, and thus resulting in the softening of Raman G peak [44].

**Figure 6:**
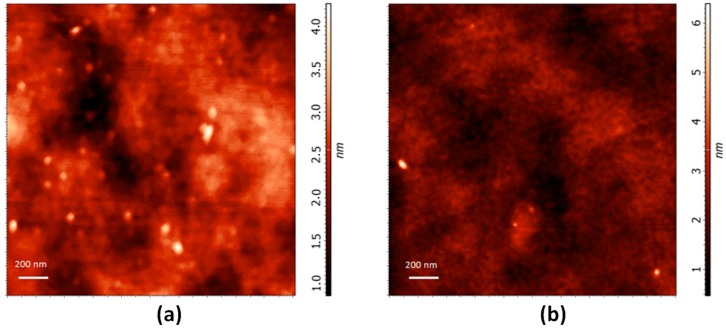
(**a**) AFM image (scan area 2 µm × 2 µm) of the GQD sample. (**b**) AFM image (scan area 2 µm × 2 µm) of the GQD-PEG-BFG@Pyr-RF sample.

The thickness distributions of GQD and of the functionalized sample GQD-PEG-BFG@Pyr-RF were characterized by atomic force microscopy. The AFM images, reported in Figure 6a,b, show that GQD are isolated and well dispersed on a Si substrate. The grains analysis shows an average height of about 2 nm for 55% grains for the GQD, while the 63% of the grains observed in the functionalized sample is characterized by an average height of about 3.681 nm. The obtained height values confirm that the functionalization processes performed on the GQD did not alter their characteristic chemical and physical properties.

**Figure 7:**
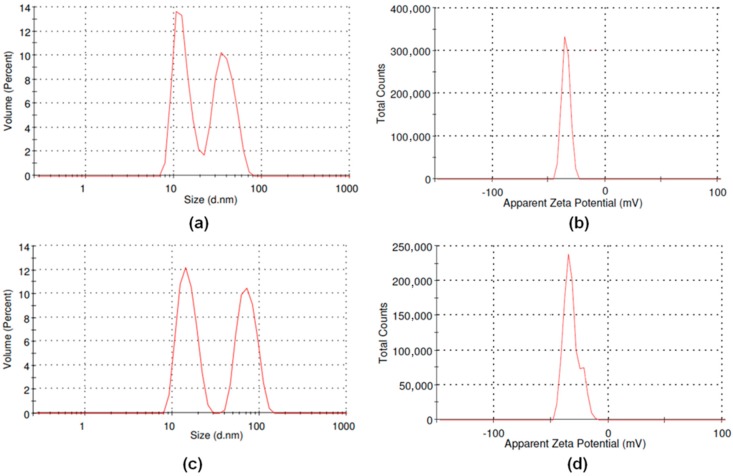
(**a**) Volume-weighted size distribution of GQD-PEG-BFG sample; (**b**) zeta potential measurement of GQD-PEG-BFG sample; (**c**) volume-weighted size distribution of GQD-PEG-BFG@Pyr-RF sample; (**d**) zeta potential measurement of GQD-PEG-BFG@Pyr-RF sample. All the experiments were performed in PBS solutions at pH 7.4.

We have also investigated the effect of the surface functionalization on the dimensions and the water dispersibility of the drug-conjugated samples GQD-PEG-BFG and GQD-PEG-BFG@Pyr-RF, which can deeply affect their interaction with cells. The results of volume-weighted size distribution, for both the samples, show two size populations with dimensions less than 100 nm (Figure 7a,c). The GQD-PEG-BFG sample shows two populations centered at 12.6 nm (51%) and 38.5 nm (49%) while for GQD-PEG-BFG@Pyr-RF, two populations centered at 14.9 (52%) and 73.1 nm (52%) can be observed. The water dispersibility was evaluated by calculating the electrophoretic mobility in PBS at the physiological pH of 7.4. The DLS analyses of GQD-PEG-BFG and GQD-PEG-BFG@Pyr-RF samples show zeta potential values of −32.89 and −31.27 mV, respectively (Figure 7b,d). The obtained values, which were always lower than −30, further confirm the high water stability of the drug conjugated systems.

### 3.4. Cytotoxicity of GQD, GQD-PEG-BFG, GQD@Pyr-RF, GQD-PEG-BFG@Pyr-RF (Red Line), and Pyr-RF in Cancer Cells

**Figure 8:**
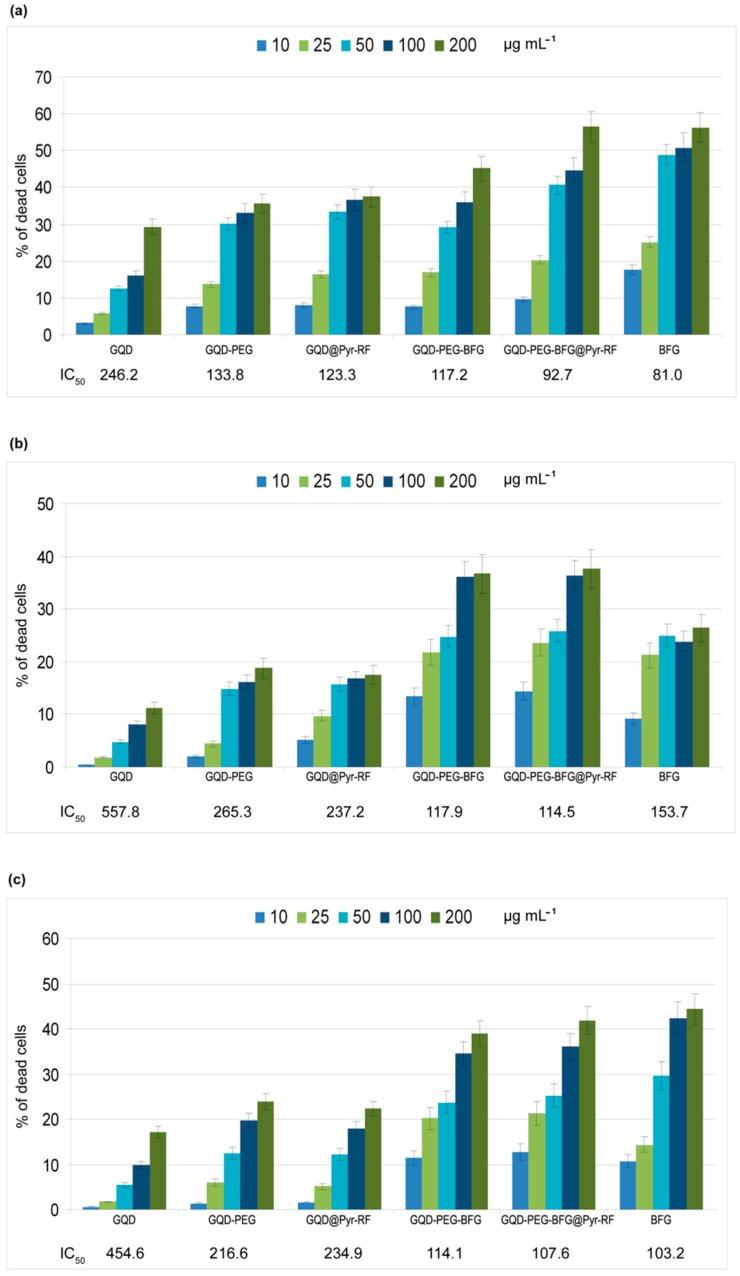
Cytotoxic effect of the synthesized drug delivery nanosystems in (**a**) laryngeal cancer cells HEp-2, (**b**) human lung epithelial cancer cell line A549, and (**c**) the human colorectal adenocarcinoma cell line HT-29. The results, obtained in cells treated for 24 h, are reported as percentages of dead cells (treated/untreated cultures). The concentrations of 10, 25, 50, 100, and 200 µg mL^−1^, shown at the top of the graphs, are those of GQD-PEG-BFG@Pyr-RF, corresponding to 2.1, 5.25, 10.5, 21, and 42 μg mL^−1^ doses of BGF assayed in the positive controls. For the GQD-PEG-BFG, the assayed doses were calculated to assess the same drug concentrations while for GQD, GQD-PEG, and GQD@Pyr-RF, the examined doses were 10, 25, 50, 100, and 200 μg mL^−1^. Each value represents the mean (±SD) of the experiments made in triplicate for each cell line. In each graph, the corresponding values of IC_50_ are reported below.

The cytotoxic effect of the synthesized GQD-based nanosystems was tested on three different cancer cell lines: laryngeal cancer cell line (HEp-2), human lung epithelial cancer cell line (A549), and human colorectal adenocarcinoma cell line (HT-29). The cytotoxicity of the drug delivery system coupled with the targeting molecule RF and conjugated with the anticancer agent BFG was compared with that exerted by the same system lacking the RF ligand (GQD-PEG-BFG) or lacking the anticancer drug (GQD@Pyr-RF) and with the reference compounds, GQD, GQD-PEG and BFG (Figure 8). The cells, maintained in the same biological medium and incubated with PBS or BFG solutions, were used as negative and positive controls, respectively. The BFG solutions used for the biological tests were prepared at a concentration similar to that of the drug present in the GQD-based samples, as evaluated by TGA analysis (range 2.1–42 mg/mL), in order to compare the drug of same concentrations for all the tested samples.

The results of biological tests underline the low cytotoxicity of the GQD sample since the percentage of dead cells in comparison to the untreated control cells at the higher tested dosages were 29% for HEp-2, 11% for A549, and 17% for HT-29 (showing IC_50_ values of 246.2, 557.8, and 454.6 µg mL^−1^, respectively). In all the assayed cell lines, the IC_50_ of the nanosystems lacking the anticancer drug, GQD-PEG and GQD@Pyr-RF samples, was approximately halved in comparison to GQD. Most likely, these increased cytotoxicities (*P* < 0.01) were due to the improved interactions between cell membranes and the chemical counterparts present in the PEG and RF moieties, thus underlining the most marked bioavailability of the assayed nanosystems. 

The drug delivery system, GQD-PEG-BFG@Pyr-RF, shows cytotoxic activity against the three investigated cancer cell lines with potency significantly higher in alveolar cancer cells compared to BFG alone (IC_50_ = 114.5 *vs.* 153.7; *P* < 0.05). In the other two cell lines (HEp-2 and HT-29), however, no significant differences were observed with respect to the drug alone, as shown by IC_50_ values that were almost superimposable. Similar behavior can be observed for the drug conjugated sample GQD-PEG-BFG lacking the targeting ligand which seems to exert cytotoxic effect preferentially in the HEp-2 cell line. 

Further biological studies performed on breast, prostate and cervical cancer cells in which RF receptors will preliminarily be quantified could, of course, clarify this issue. In the present study, the cytotoxicity tests highlighted how despite the low water solubility of BFG alone, the drug-conjugated nanosystem shows enhanced dispersibility in water and improved cell-drug interactions, thus opening new possibilities in the use of this or other anticancer drugs poorly soluble in water and endowed of cytotoxicity and critical side effects.

## 4. Conclusions

In this study, we report the synthesis of a smart DDS, based on GQD, for anticancer therapy. The possibility offered by the multimodal conjugation of these nanomaterials allowed the introduction of an anticancer drug by means of a PEG linker and of the targeting ligand riboflavin by π–π interaction, using a pyrene linker. 

The evaluation of the cytotoxicity, tested on three different cancer cell lines, underlined the low cytotoxicity of the GQD and the ability of the DDS to enhance interactions between the anticancer drug and the cancer cells causing a cytotoxic effect with a higher potency compared to that exerted by the drug alone in the human lung epithelial cancer cell line. The improved dispersibility of the nanosystem in water with respect to the anticancer drug alone leads to an improved pharmacological profile after its conjugation with the GQD. 

The reported results represent another step towards more innovative targeted therapies for cancer treatment, opening new possibilities in the use of anticancer drugs poorly soluble in water and endowed with low cell uptake, systemic toxicity, and undesirable side effects.

## Supplementary Materials

The following are available online at http://www.mdpi.com/2079-4991/9/2/282/s1, Figure S1: XRD spectra of MWCNT and GQD, Figure S2: UV–vis absorption spectrum of GQD, Figure S3: PL spectra of GQD dispersion in deionized water, Figure S4: TGA of PEG-NH_2_ and of BFG; Figure S5: ^1^HNMR data and spectrum of Pyr-RF sample, Figure S6: HRMS data and spectrum of Pyr-RF sample, Figure S7: TGA of GQD, GQD@Pyr-RF, GQD-PEG-BFG and GQD-PEG-BFG@Pyr-RF.
