# Supplementary material for: A Smart Nanovector for Cancer Targeted Drug Delivery Based on Graphene Quantum Dots
Source: Nanomaterials (Basel). 2019 Feb 18;9(2):282. doi: 10.3390/nano9020282 (PMC6409783; doi:10.3390/nano9020282)
Supplement: Supplementary file 1 [file nanomaterials-09-00282-s001.pdf]

Supporting Information to

A smart nanovector for cancer targeted drug delivery based on graphene quantum dots

Daniela Iannazzo,[a*] Alessandro Pistone,[a*] Consuelo Celesti,[a] Claudia Triolo,[b] Salvatore Patanè,[b] Salvatore V. Giofré,[c] Roberto Romeo,[c] Ida Ziccarelli,[d] Raffaella Mancuso,[d] Bartolo Gabriele,[d] Giuseppa Visalli,[e] Alessio Facciolà[e] and Angela Di Pietro[e]

[a] *Department of Engineering, University of Messina, Contrada Di Dio, I-98166 Messina, Italy*

[b] *Department of Mathematical and Computer Sciences, Physical Sciences and Earth Sciences, Viale F. Stagno d'Alcontres, 98166 Messina, Italy*

[c] *Department of Chemical, Biological, Pharmaceutical and Environmental Sciences, University of Messina, Viale Annunziata, I-98168 Messina, Italy*

[d] *Laboratory of Industrial and Synthetic Organic Chemistry (LISOC), Department of Chemistry and Chemical Technologies, University of Calabria, Via Pietro Bucci 12/C, 87036 Arcavacata di Rende (CS), Italy*

[e] *Department of Biomedical and Dental Sciences and Morphological and Functional Images, University Hospital of Messina, Via Consolare Valeria, 1, 98100, Messina, Italy*

[*]Corresponding authors.

E-mail addresses: diannazzo@unime.it; pistone@unime.it; Fax: +39 090 6765494; Tel: +39 090 6765569

Table of Contents

Figure S1. XRD spectra of MWCNT and GQD……………………………………….....……....S2

Figure S2. UV−vis absorption spectrum of GQD……………………………………….……....S2

Figure S3. PL spectra of GQD dispersion in deionized water ….…………………….……….……S3

Figure S4. TGA of PEG-NH_2 and of BFG……………………………………..………………S3

Figure S5. ^{1}HNMR data and spectrum of Pyr-RF sample……………………………............S4

Figure S6. HRMS data and spectrum of Pyr-RF sample……………………………………..S4

Figure S7. TGA of GQD, GQD@Pyr-RF, GQD-PEG-BFG and GQD-PEG-BFG@Pyr-RF........S5

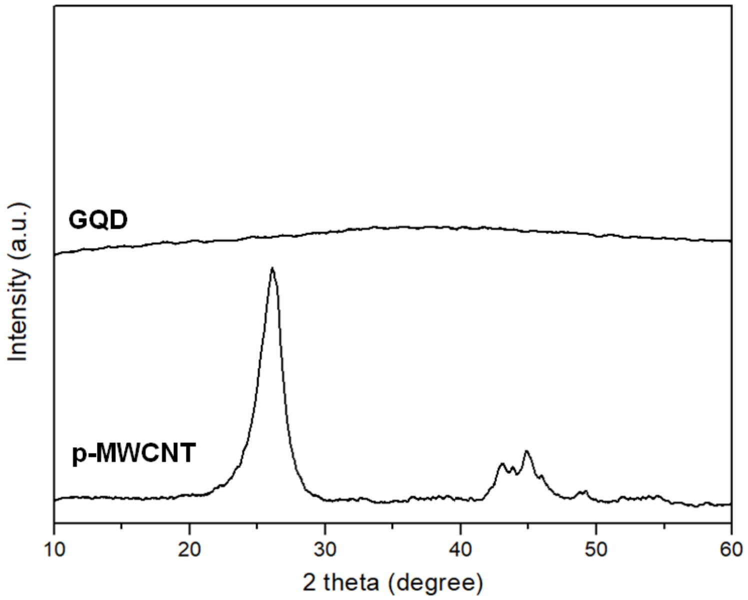

Figure S1. XRD spectra of GQD and of pristine MWCNT(p-MWCNT).

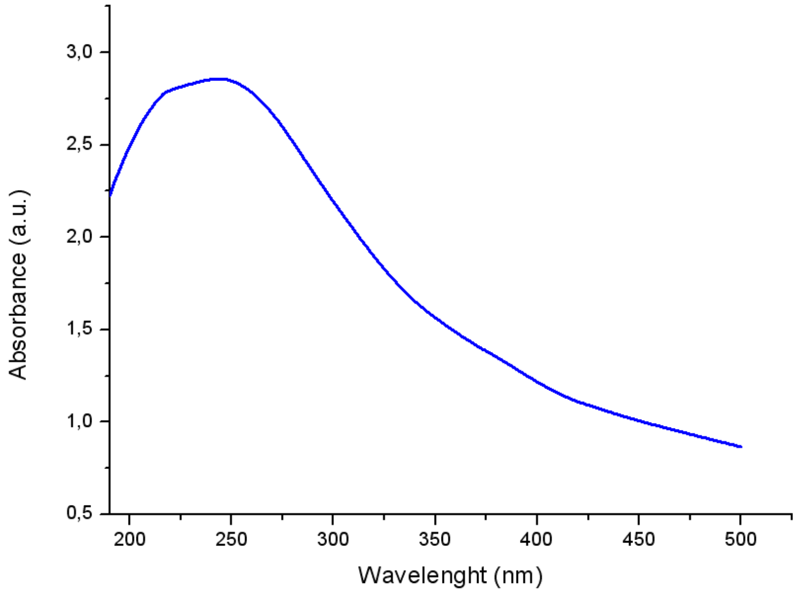

Figure S2. UV−vis absorption spectrum of GQD in deionized water.

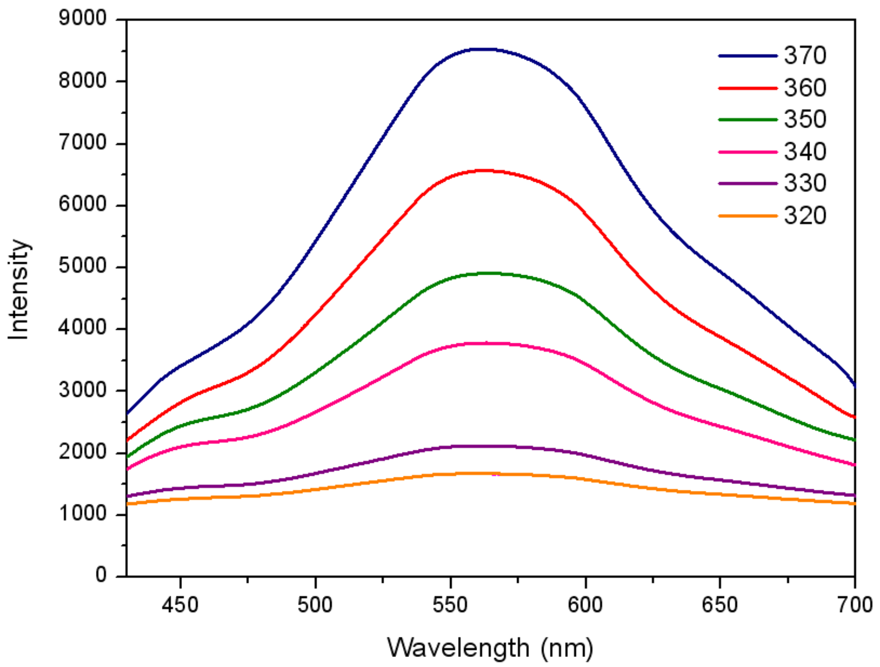

Figure S3. PL spectra of GQD dispersion in deionized water at the excitation wavelengths of 320, 330, 340, 350, 360 and 370 nm.

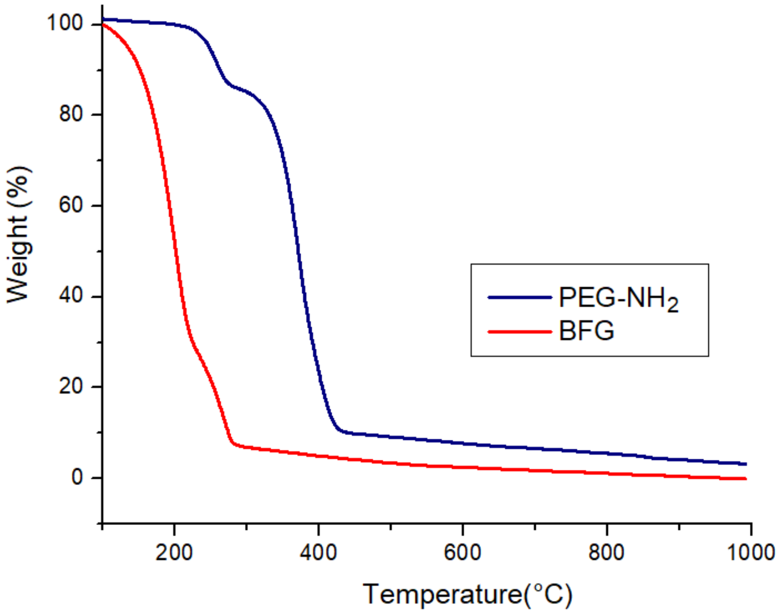

Figure S4. TGA curves for $PEG-NH_2$ and of BFG. All experiments were performed under argon atmosphere.

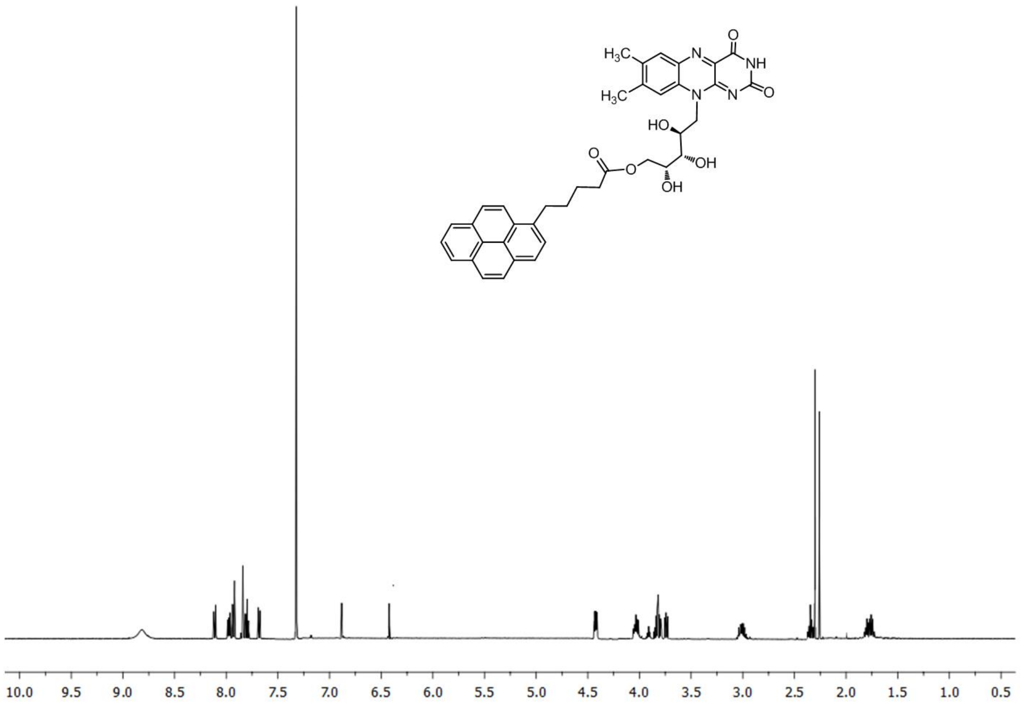

Figure S5. ^{1}H NMR spectrum of Pyr-RF sample. ***5-(7,8-dimethyl-2,4-dioxo-3,4-dihydrobenzo[g]pteridin-10(2H)-yl)-2,3,4-trihydroxypentyl-5-(pyren-1-yl)pentanoate.*** ^{1}H NMR (300 MHz, $CDCl_3$) δ = 8.72 (br s, 1H), 8.12 (d, 1H, J = 7.4 Hz), 7.83-7.68 (m, 9H), 6.85 (s, 1H), 6.40 (s, 1H), 4.46 (dd, 1H, J = 11.92, 6.8 Hz), 4.08-3.89 (m, 2H), 3.87-3.83 (m, 1H), 3.80-3.75 (m, 3H), 3.70-3.66 (m, 2H), 3.06-2.98 (m, 2H), 2.36-2.33 (m, 5H), 2.26 (s, 3H), 1.75-1.68 (m, 4H).

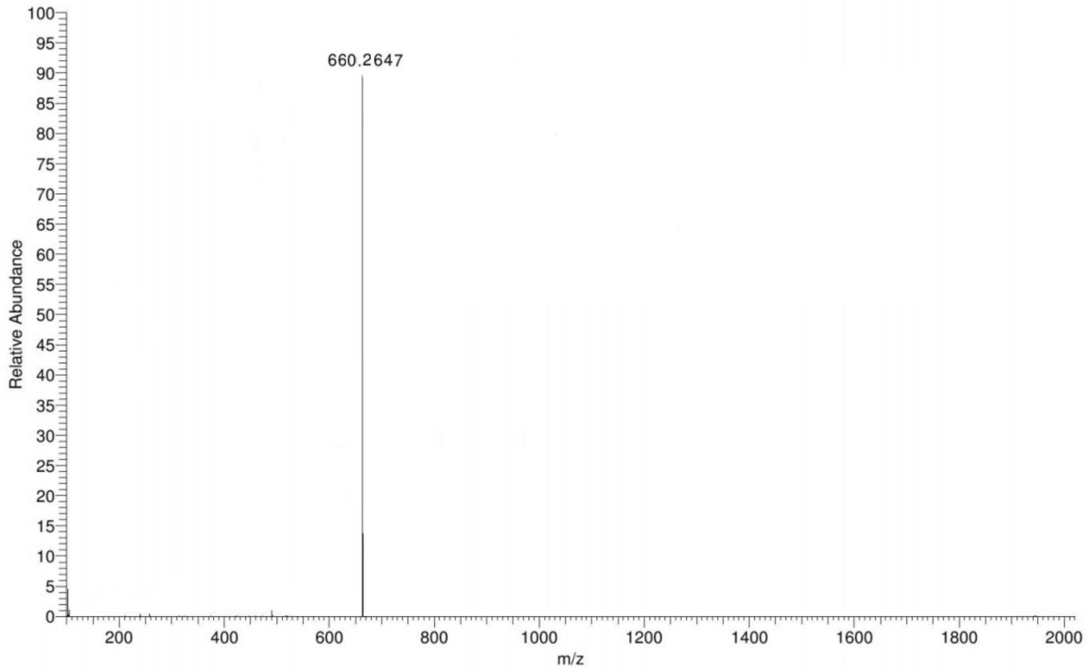

Figure S6. HMRS spectrum of Pyr-RF sample. HRMS (EI) for (M^{+}) $C_{38}H_{36}N_4O_7$, calcd 660.2645, found 660.2647.

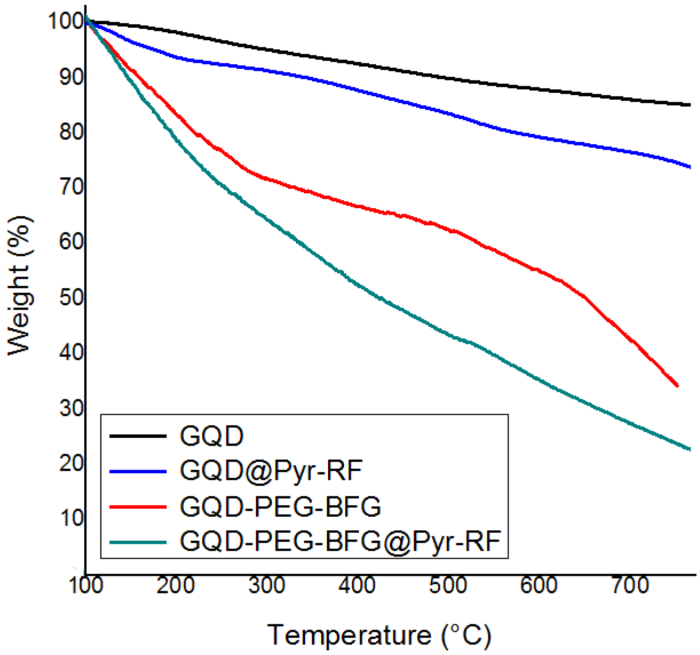

Figure S7. TGA curves for GQD, GQD@Pyr-RF, GQD-PEG-BFG and GQD-PEG-BFG@Pyr-RF. All experiments were performed under argon atmosphere.
